# Supplementary material for: Dissecting the cellular specificity of smoking effects and reconstructing lineages in the human airway epithelium
Source: Nat Commun. 2020 May 19;11:2485. doi: 10.1038/s41467-020-16239-z (PMC7237663; doi:10.1038/s41467-020-16239-z)
Supplement: Supplementary file 1 — Supplementary Information [file 41467_2020_16239_MOESM1_ESM.pdf]

Supplementary Information for:

Dissecting the cellular specificity of smoking effects and reconstructing lineages in the human airway epithelium

Authors/Affiliations

Katherine C. Goldfarbmuren[1#], Nathan D. Jackson[1#], Satria P. Sajuthi[1], Nathan Dyjack[1], Katie S. Li[1], Cydney L. Rios[1], Elizabeth G. Plender[1], Michael T. Montgomery[1], Jamie L. Everman[1], Preston E. Bratcher[2,3], Eszter K. Vladar[4,5], Max A. Seibold[1,2,4,*]

[1]Center for Genes, Environment, and Health, National Jewish Health, Denver, CO, 80206 USA; [2]Department of Pediatrics, National Jewish Health, Denver, CO, 80206 USA; [3]Department of Pediatrics, [4]Division of Pulmonary Sciences and Critical Care Medicine and [5]Department of Cell and Developmental Biology, University of Colorado-AMC, Aurora, CO, 80045 USA, [#]these authors contributed equally to this work, *correspondence: seiboldm@njhealth.org

Supplementary Fig. 1

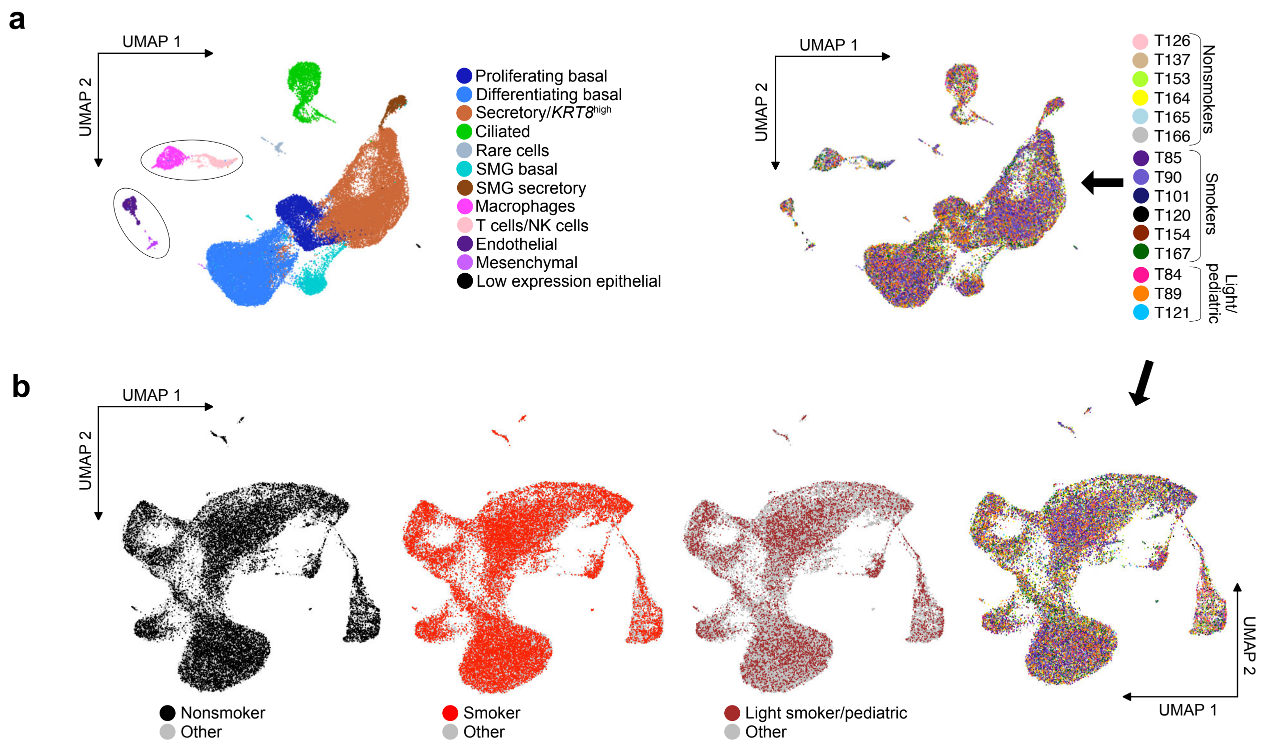

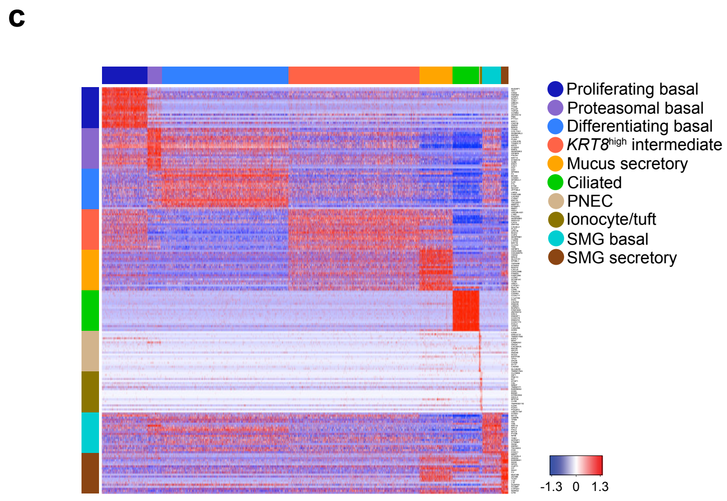

d

	Donor	Ionocyte	Tuft	PNEC
Nonsmokers	T126	0.0017	0.0067	0.0017
	T137	0.0000	0.0015	0.0000
	T153	0.0035	0.0044	0.0015
	T164	0.0018	0.0015	0.0028
	T165	0.0009	0.0038	0.0019
	T166	0.0065	0.0011	0.0030
Smokers	T85	0.0061	0.0008	0.0029
	T90	0.0027	0.0040	0.0009
	T101	0.0042	0.0058	0.0021
	T120	0.0011	0.0005	0.0027
	T154	0.0018	0.0000	0.0030
	T167	0.0028	0.0010	0.0056
Light/pediatric	T84	0.0016	0.0011	0.0037
	T89	0.0004	0.0037	0.0015
	T121	0.0007	0.0000	0.0007

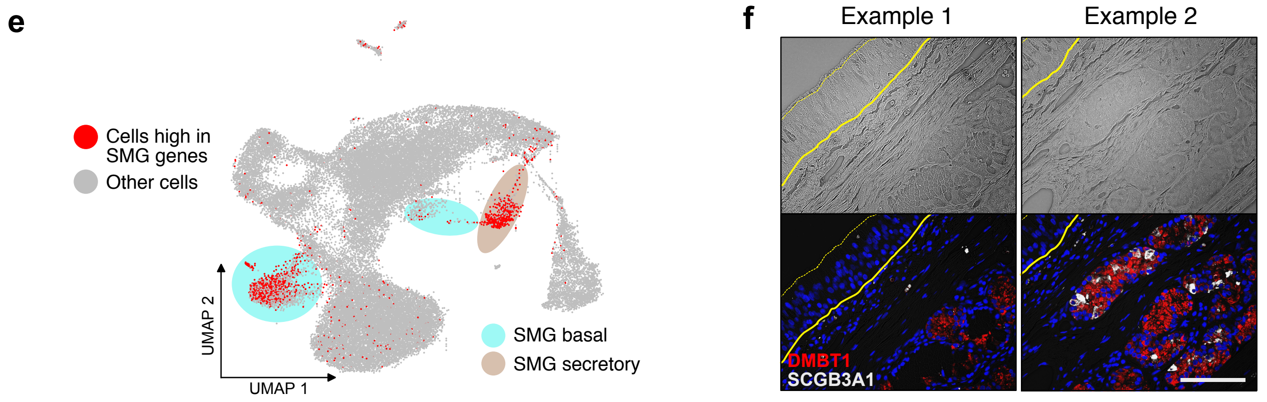

Supplementary Fig. 1 (with Fig. 1): Full *in vivo* scRNA-seq analysis

- **a.** UMAP of original *in vivo* dataset including four non-epithelial clusters (circled) excluded from further analysis. *Left,* Coloring corresponds to cell types based on SNN clustering. *Right,* Coloring corresponds to donor/smoke status.
- **b.** UMAP of epithelial cell clusters inferred after removing non-epithelial clusters. *Left,* Coloring corresponds to smoke status. *Right*, Coloring corresponds to donor as indicated in legend in **a**.
- **c.** Heat map depicts 20 genes (y-axis) distinguishing each of the broad cell populations in the human trachea. Cells are distributed along the x-axis (see Fig. 1).
- **d.** Proportion of each rare cell population relative to total cells across all donors.
- **e.** Cells (in red) both highly expressing SMG markers (>75th quantile mean expression) and lowly expressing epithelial surface markers (< 25th quantile mean expression) from Fischer et al. (2007)[1] were strongly enriched in basal and secretory SMG populations *in vivo*. SMG and surface markers constituted the top 50 of those most upregulated in human bronchial SMG and surface tissue, respectively, when transcriptionally compared to one another.
- **f.** Additional IF labeling of SMG markers, DMBT1 and SCGB3A1. Upper panels, transillumination; solid yellow line, basement membrane; dashed yellow line, luminal surface of the epithelium. Scale bar is 100 μm.

Supplementary Fig. 2

a

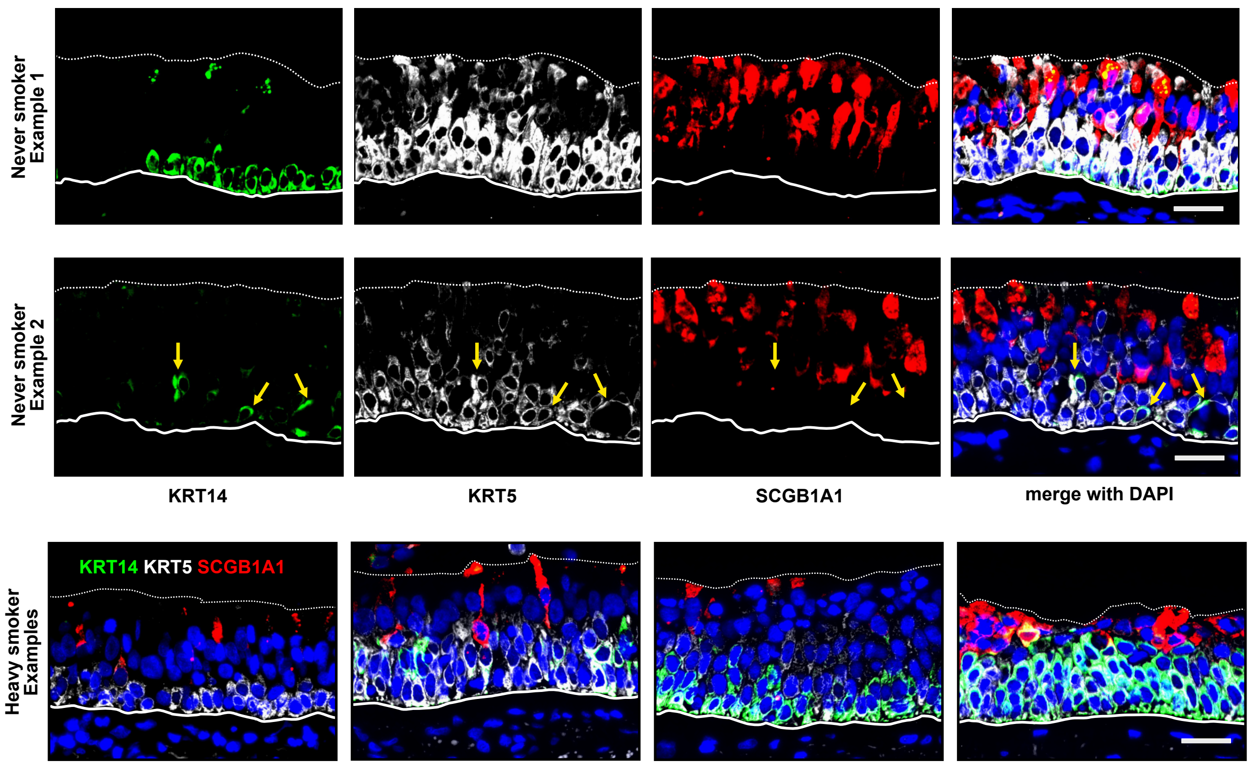

b

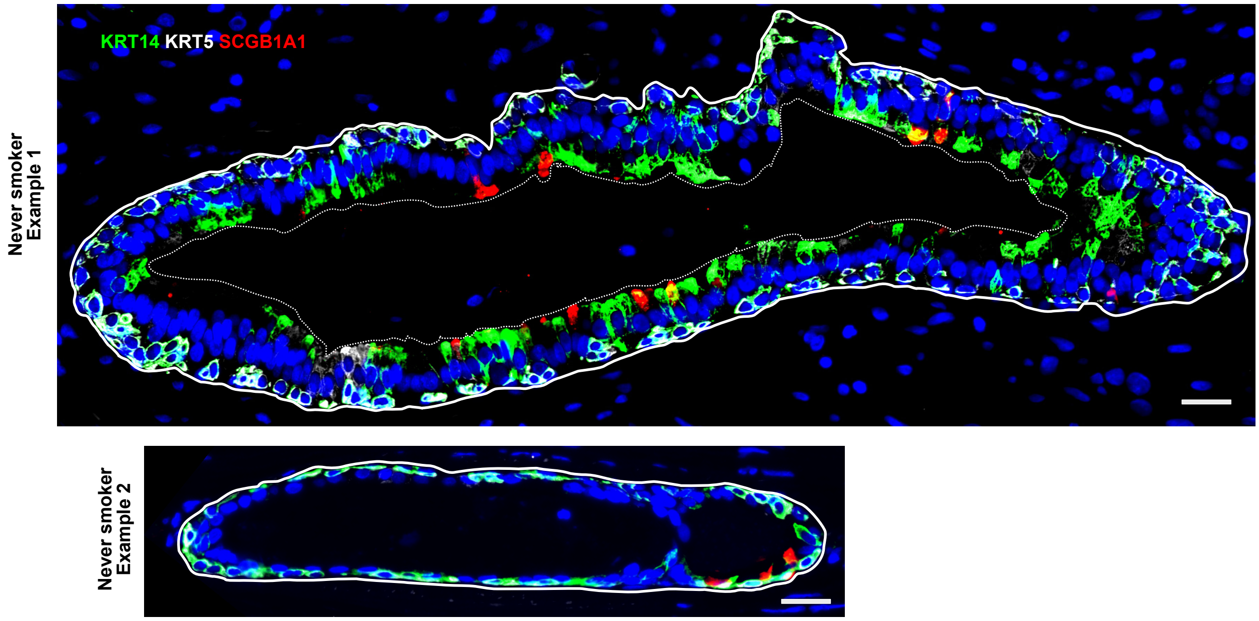

Supplementary Fig. 2 (with Fig. 1): KRT14 in the human airway epithelium

- **a.** *Top* and *Middle,* Representative examples of IF labeled human tracheal epithelium from never smokers illustrate KRT14 (green) in a subset of KRT5+ (white) basal cells. SCGB1A1 is in red, DAPI in blue, dashed and solid lines represent the apical edge and basement membrane of the epithelium, respectively. Yellow arrows highlight select KRT14+ cells to aid assessment in the other channels. Scale bar = 25 μm. *Bottom*, Representative examples of IF labeled human tracheal epithelium from smokers indicate a wide range of KRT14 (green) presence and depleted SCGB1A1 (red) relative to never smokers. Scale bar = 25 μm.
- **b.** Representative examples of IF labeled human tracheal SMG from never smokers demonstrate KRT14 (green) in nearly all basal cells as well as some more luminally oriented cells. KRT5 is in white, SCGB1A1 in red, DAPI in blue, dashed line represents the apical side of the glandular epithelium. Scale bar = 25 μm.

Supplementary Fig. 3

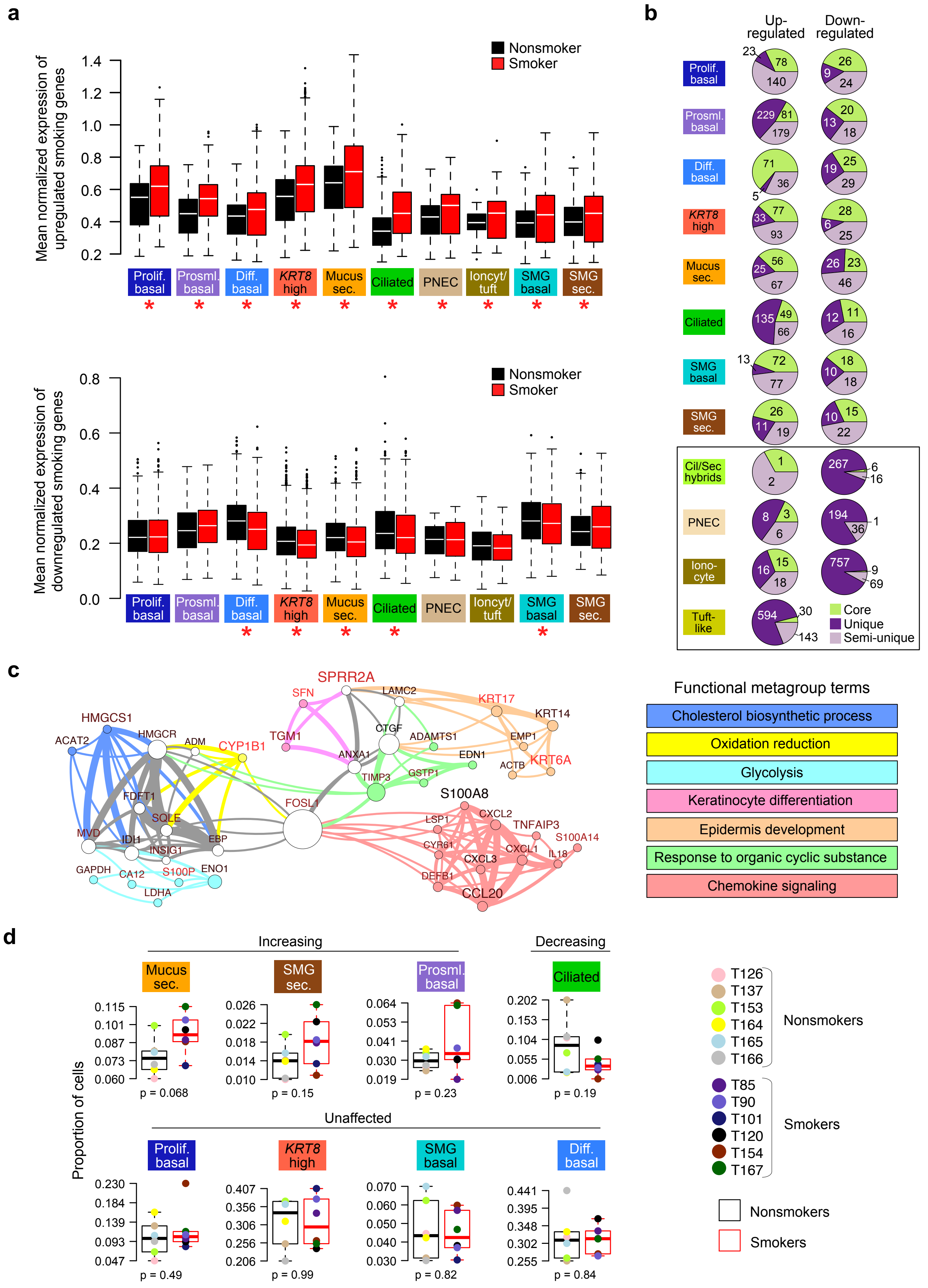

Supplementary Fig. 3 (with Fig. 2): Shared and unique smoking responses across diverse epithelial cell types

- **a.** *Top*, Upregulated smoking genes from bulk RNA-seq[2] were upregulated in smokers compared to nonsmokers in all single cell populations, whereas, *Bottom*, downregulated smoking genes[2] were suppressed in half of the single cell populations. Box plots show the geometric mean of normalized gene expression across cells within each population. There were 120 genes (of 130 reported) or 48 genes (of 55 reported) expressed, and thus averaged over, in the single cell dataset for up- or downregulated bulk smoking gene sets, respectively. *p-value < 0.05 based on one-sided t-tests comparing means for smokers and nonsmokers (exact p-values for each comparison, left to right, top: 2.15e-58, 5.04e-33, 1.57e-70, 2.76e-117, 5.39e-24, 2.52e-68, 0.0210, 0.00313, 9.75e-13, 0.0116; bottom: 0.468, 0.990, 2.26e-69, 1.81e-21, 1.43e-7, 1.38e-4, 0.494, 0.105, 0.00496, 0.950). 53 (44%) and 9 (19%) of up- and downregulated bulk smoking genes were up- or downregulated, respectively, by smoking in our dataset in at least 1 population (including rare cells). Furthermore, up and downregulated smoking response genes in all main cell populations were significantly enriched for the up and downregulated bulk smoking response genes, respectively (one-sided Fisher exact test p-value < 0.05), except for the upregulated genes for the SMG secretory population. For box plots, box centers give the median, upper and lower box bounds correspond to first and third quartiles, and the upper/lower whiskers extend from the upper/lower bounds up to/down from the largest/smallest value, no further than 1.5 x IQR from the upper/lower bound (where IQR is the inter-quartile range). Data beyond the end of whiskers are plotted individually.
- **b.** Pie charts showing proportions of genes significantly up or downregulated with smoking for each cluster that were core, unique, or semi-unique. Raw numbers of genes in each category are given in the plots. Core genes were defined as those up in at least five of the eight main non-rare cell populations (the top 8 populations shown). Unique and semi-unique (i.e., non-core yet non-unique) genes were defined for these same eight populations in respect to one another (see also Fig. 2a). For the four subgroups in the bottom box, core genes were the same as inferred above and unique and semi-unique genes were defined in respect to the combined set of eight main populations and four subgroups.
- **c.** Functional gene network (FGN) of core smoking response genes that were upregulated in five or more populations summarizes the genes and functional pathways activated across epithelial cell types. Edges connect genes annotated

for the same enrichment terms. Node colors denote functional categories (or metagroups) to which genes belong. Exemplar terms for each metagroup are given on the right. White nodes indicate genes belonging to more than one metagroup (hub genes). Node size indicates the connectivity of genes within the network and gene label size or redness increase with increasing mean log fold change or increasing mean significance, respectively, of smoking DEGs. Edge thickness increases with the number of shared terms and edge color indicates metagroup membership of the connected node(s) if one or both are not hub genes. Grey edges connect two hub genes.

d. Box plots depicting the proportion of total cells from each donor belonging to each of the indicated cell clusters, stratified by smoking status (n = 6 smokers and 6 nonsmokers; see also Fig. 2c)**.** Underlying distributions for box plots are as described in **a**. P-values are based on two-sided t-tests comparing means for smokers and nonsmokers.

Supplementary Fig. 4

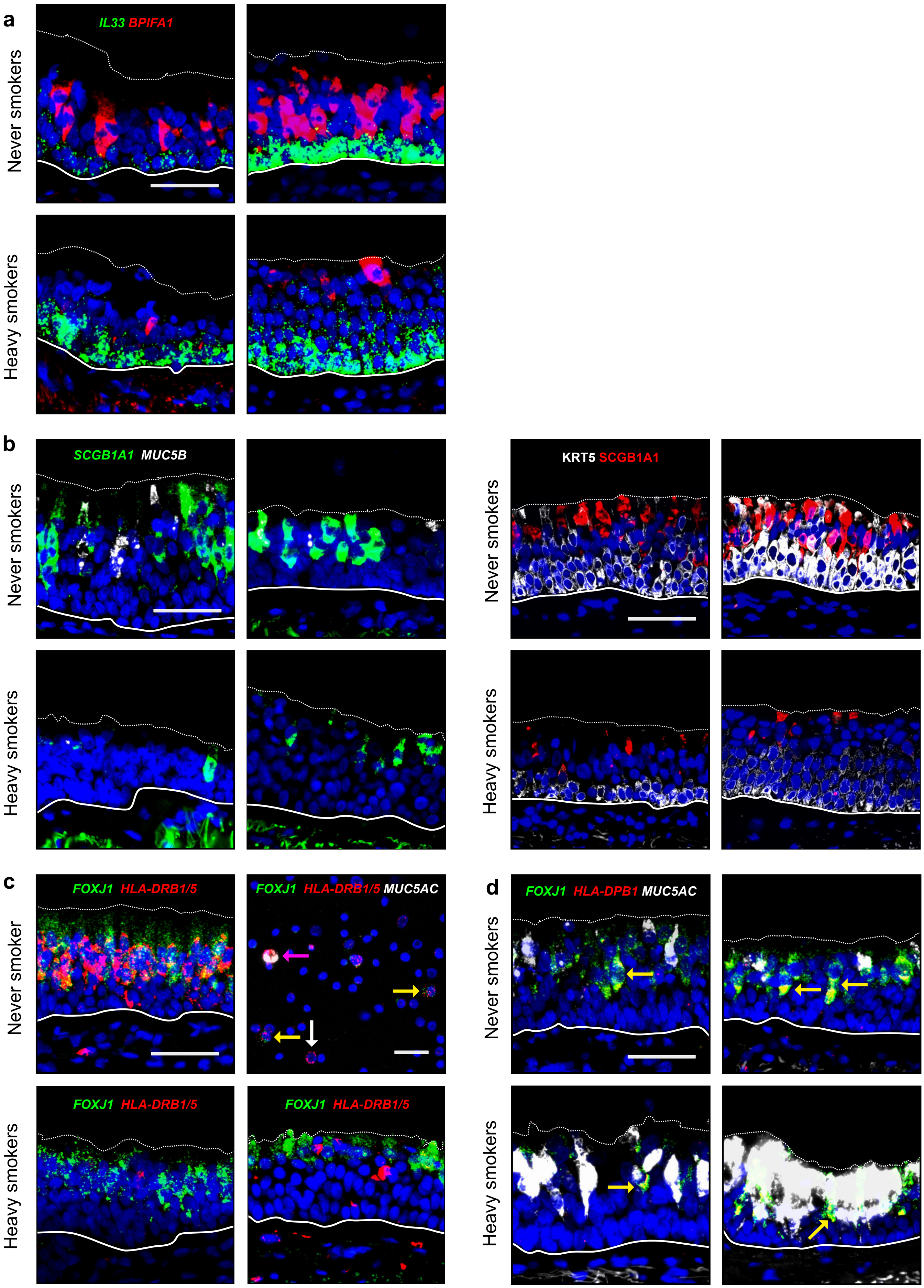

Supplementary Fig. 4 (with Fig. 2): Representative histological images support scRNA-seq smoking response and HLA mRNA expression by the human airway epithelium

- **a.** *BPIFA1* mRNA appears depleted in heavy smokers (representative images from 4 fields each of 2 never or heavy smokers). Scale bar = 25 µm.
- **b.** *Left, SCGB1A1* mRNA and *Right,* SCGB1A1 protein appear depleted in heavy smokers (representative images from at least 8 fields each of 2 never or heavy smokers). Scale bar = 25 µm.
- **c.** *HLA-DRB1/5* mRNA is present in epithelial cells and appears depleted in heavy smokers. *Top left,* Representative tissue section from never smoker shows epithelial mRNA localization (white). *Top right,* cytospin from never smokers shows co-cellular localization of *HLA-DRB1/5* (in white) with epithelial cell markers *FOXJ1* (in green, yellow arrows) or *MUC5AC* (in red, pink arrow). *White arrow*, *HLA-DRB1/5* without epithelial markers. *Bottom, HLA-DRB1/5* appears depleted in heavy smokers (representative images from 4 fields each of 2 heavy smokers). Scale bars = 25 µm.
- **d.** *HLA-DPB1* mRNA co-localizes with *FOXJ1* mRNA and *MUC5AC* mRNA expression appears to increase in heavy smokers (representative images from 5 fields each of 2 never or heavy smokers). Scale bar = 25 µm.

Supplementary Fig. 5

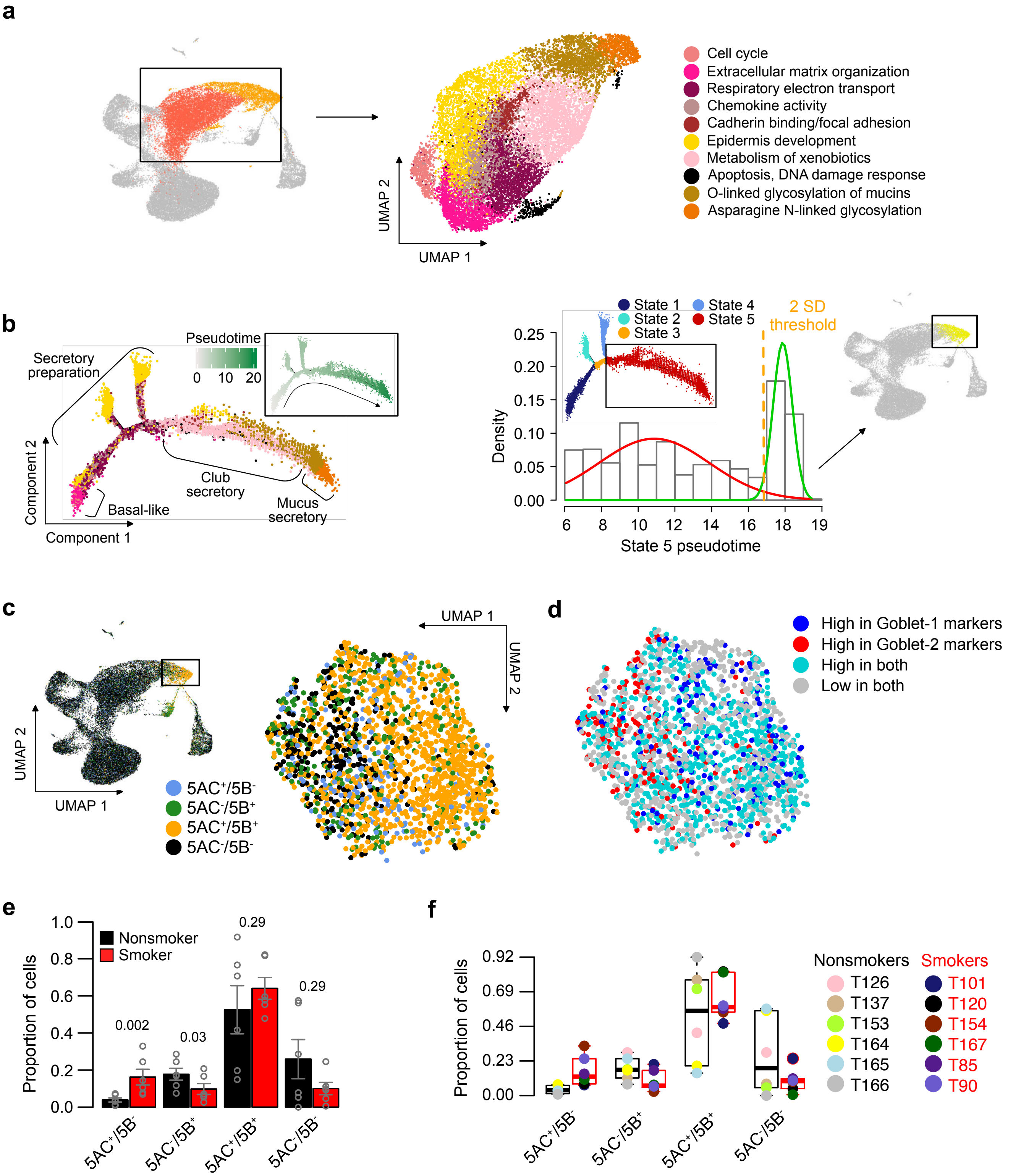

Supplementary Fig. 5 (with Fig. 3): *In vivo* secretory cell lineages and smoking effects

- **a.** *Left*, UMAP depicting subclustering of *KRT8*^{high} and mucus secretory cells. Colors correspond to distinct SNN subclusters. *Right*, Representative top enriched pathways for each subcluster based on differentially expressed genes.
- **b.** *Left*, Monocle pseudotime trajectory[3] of *KRT8*^{high} and mucus secretory cells. Colors correspond to subclusters in **a**, revealing that cell ordering by Monocle is broadly concordant with UMAP coordinates. Bracketed pseudotime labels indicate the approximate location of four major expression phases (see Fig. 3ab). *Left inset*, the direction of pseudotime, assuming that the root state corresponds to the most basal-like cells in the trajectory. *Right*, Mixed Gaussian density curves fitted to the distribution of pseudotime values for cells belonging to the final state (state 5) along the trajectory. To isolate fully differentiated secretory cells at the end of pseudotime from those differentiating cells along the branch leading to these cells (which we assumed would exhibit distinct pseudotime distributions), we selected those cells falling within at least two standard deviations of the second distribution.
- **c.** UMAP depicting a lack of distinct subtypes within the *in vivo* human mucus secretory cells, although a continuum of increasing mucin expression is evident along dimension 1. Mucin co-expression status as in Fig. 3c.
- **d.** Although average expression of goblet cell 1 (“goblet-1”) and goblet cell 2 (“goblet-2”) subtype markers reported in Montoro et al. (2018)[4] does show slight correspondence to level of mucin transcription in mature mucus secretory cells (compare to UMAP in **c**), these subtypes do not strongly distinguish human tracheal secretory cells *in vivo*. “High” and “low” expressing cells are defined as those with average expression above or below the 50% percentile, respectively.
- **e.** Bar plots showing that *MUC5AC*-only expressing mucus secretory cells increase in prevalence with smoking. Furthermore, double negative and *MUC5B*-only cells tend to acquire *MUC5AC* expression with prolonged exposure to smoke. P-values from one-sided Wilcox tests comparing smokers and nonsmokers in each mucin co-expression class are indicated above each plot. N=6 donors for each smoke status. Error bars show standard error for cell proportions and points give the proportions for each donor.
- **f.** Box plots showing cell frequencies for the same mucin co-expressing panels as in **e** (n = 6 nonsmoker donors and 6 smoker donors). Box centers give the median, upper and lower box bounds correspond to first and third quartiles, and the upper/lower whiskers extend from the upper/lower bounds up to/down from

the largest/smallest value, no further than 1.5 x IQR from the upper/lower bound (where IQR is the inter-quartile range). Data beyond the end of whiskers are plotted individually.

Supplementary Fig. 6

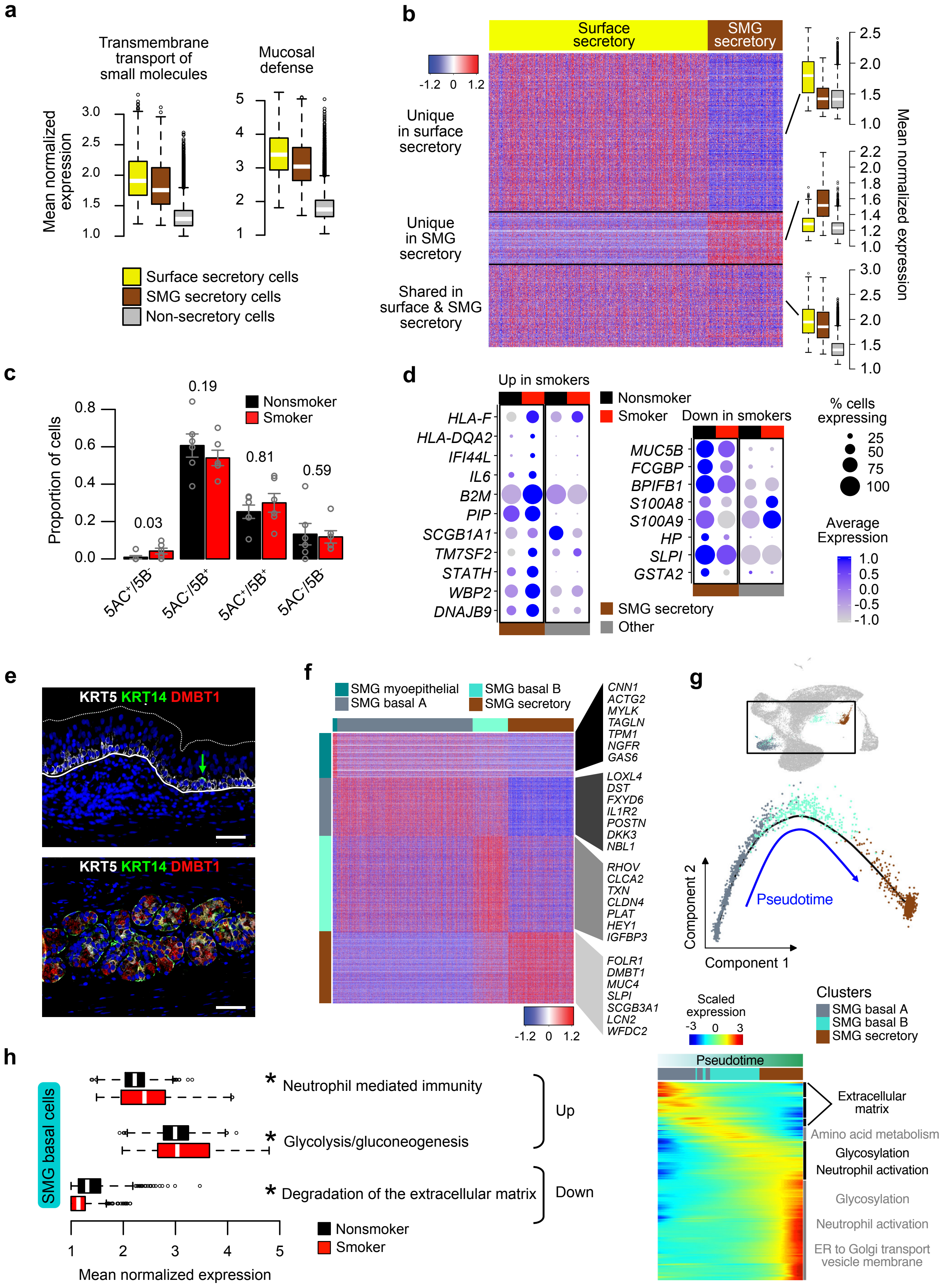

Supplementary Fig. 6 (with Fig. 4): Human submucosal gland - distinctions from the surface epithelium, lineages, and smoking effects

- **a.** Box plots show mean expression across groups of genes that were upregulated in both surface epithelial (n = 1,858) and SMG secretory (n = 633) cells compared to non-secretory cells (n = 32,589). *Left*, genes were annotated for the specified enriched term. *Right*, "Mucosal defense" includes the following genes: *BPIFB1*, *BPIFA1*, *C3*, *CP*, *CD55*, *CYP2F1*, *GSTA1*, *LCN2*, *LYN*, *LYZ*, *PIGR*, *SLPI*, and *WFDC2*. Box centers give the median, upper and lower box bounds correspond to first and third quartiles, and the upper/lower whiskers extend from the upper/lower bounds up to/down from the largest/smallest value, no further than 1.5 x IQR from the upper/lower bound (where IQR is the inter-quartile range). Data beyond the end of whiskers are plotted individually.
- **b.** Heat map of all DEGs that were either unique to or shared by surface epithelial and SMG secretory cells compared to non-secretory cells. Box plots at right show average expression of each gene block in secretory and non-secretory cells in the tracheal epithelium. Sample sizes and box plot distributions are the same as in **a**.
- **c.** Bar plots comparing the proportion of cells belonging to each mucin co-expression class between nonsmokers (black; n = 6) and smokers (red; n = 6). P-values from one-sided Wilcox tests are indicated above each plot. Error bars show standard error for cell proportions and points give the proportions for each donor.
- **d.** Dot plots showing how level and ubiquity of expression change in SMG secretory cells compared to other tracheal cells, when looking at select (non-core) genes that significantly responded to smoking in these SMG secretory cells. *Left,* genes upregulated by smoking; *Right*, genes downregulated by smoking.
- **e.** Immunohistochemistry of SMG secretory and basal markers indicates that DMBT1 and KRT14 are highly specific to SMG secretory and basal cells, respectively. Green arrow in top panel indicates a lone $KRT14^+$ cell in the surface epithelium. Scale bar = 50 µm.
- **f.** Heat map of DEGs distinguishing SMG basal cell substates and mature SMG secretory cells. Select genes are indicated for each DEG block. Full gene lists are given in Source Data.
- **g.** *Top,* Monocle pseudotime trajectory[3] connecting SMG basal cell substates (excluding myoepithelial cells) to SMG secretory cells. *Bottom,* Heat map showing scaled smoothed expression of pseudotime-dependent genes across

the trajectory, assuming that SMG basal cells occupy the root state. Summary enrichment terms are shown for the indicated gene groups on the right.

h. Box plots showing mean expression across groups of smoking DEGs in SMG basal cells that were annotated for select enriched pathways. Cells were segregated by smoking status (n = 701 nonsmoker cells and 700 smoker cells). Box plot distributions are the same as in **a**. *Means of average gene group expression were significantly elevated (top two pathways) or depressed (bottom pathway) in smokers based on one-sided Wilcox tests (p-values, top to bottom: 3.46e-11, 7.56e-5, and 3.87e-42).

Supplementary Fig. 7

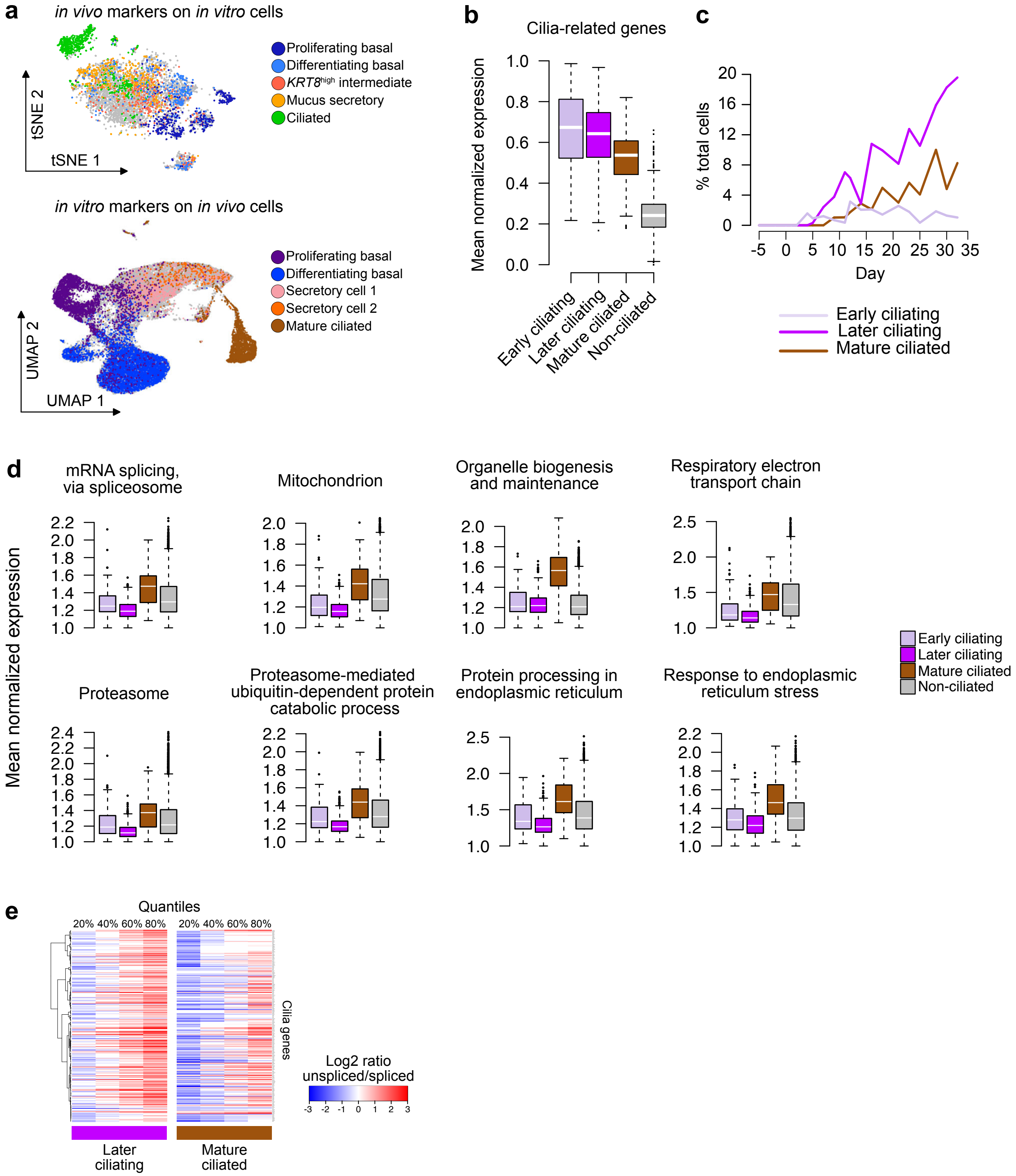

Supplementary Fig. 7 (with Fig. 5): Relations between *in vitro* and *in vivo* cell states, *in vitro* ciliated cell characterization, and splicing analysis

- **a.** Broad cell populations *in vivo* are analogous to broad cell populations *in vitro*. *Top,* characteristic expression of *in vivo* markers for each select broad cell type is overlaid onto *in vitro* tSNE cells. *Bottom,* characteristic expression of *in vitro* markers of select cell types is overlaid onto *in vivo* UMAP cells. Grey cells indicate those not characteristic of any of the broad cell types shown, based on the thresholds of expression used (see Methods).
- **b.** Signature genes from the *in vivo* mature ciliated cell population were common to the three *in vitro* ciliated cell populations (n = 66 for early ciliating, 403 for later ciliating, and 152 for mature ciliated), distinguishing them from the rest of the non-ciliated epithelium (n = 5,355). Box centers give the median, upper and lower box bounds correspond to first and third quartiles, and the upper/lower whiskers extend from the upper/lower bounds up to/down from the largest/smallest value, no further than 1.5 x IQR from the upper/lower bound (where IQR is the inter-quartile range). Data beyond the end of whiskers are plotted individually.
- **c.** Proportions of epithelial cells belonging to early ciliating, later ciliating and mature ciliated cell *in vitro* states across culture time points support the pseudotime trajectory in Fig. 5e.
- **d.** Enrichment analysis of DEGs upregulated in mature ciliated cells (n = 152) relative to later ciliating cells (n = 403) reveals that the mitochondrial maintenance and protein processing genes were particularly elevated in this population. Box plots depict mean expression of DEGs annotated for the indicated enriched terms (n = 66 early ciliating cells and 5,355 non-ciliated cells). Box plot distributions are as in **b**.
- **e.** Ratio of unspliced/spliced RNA within later ciliating and mature ciliated cells suggests that specific ciliation program genes are poised (i.e., unspliced) in the later ciliating population, but active (i.e., spliced) in the mature ciliated cells. Quantiles of cells with both spliced and unspliced expression have a higher unspliced/spliced ratio across 243 cilia genes.

Supplementary Fig. 8

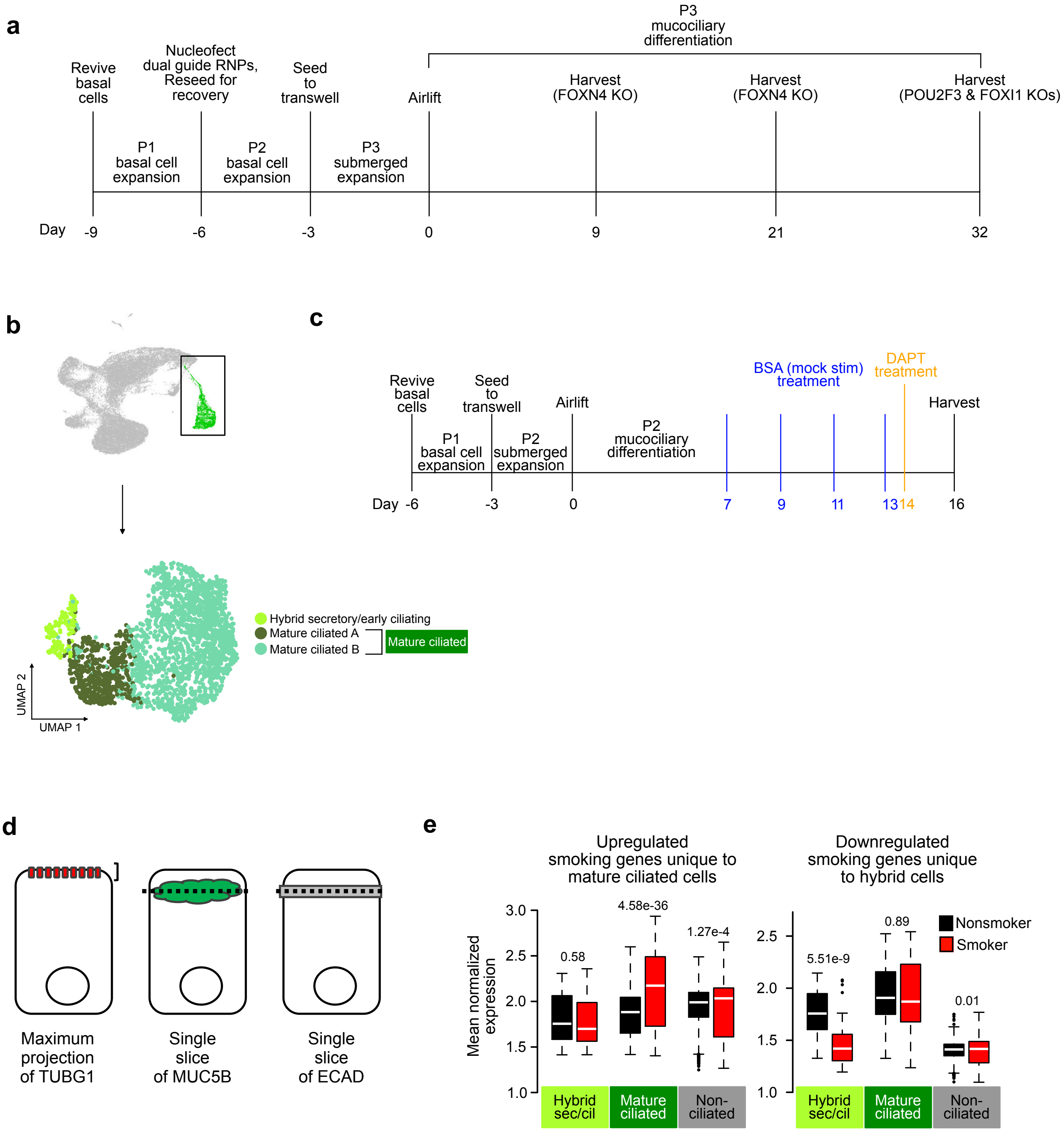

Supplementary Fig. 8 (with Fig. 6): Dual exonic CRISPR scheme, *in vivo* ciliated cell subclustering, hybrid cell validation, and smoking effects

- **a.** Schematic for CRISPR-Cas9 editing of primary human tracheal basal cells
- **b.** UMAP depicts SNN subclustering of *in vivo* ciliated cells. “Mature” subgroups A and B were combined for further analysis.
- **c.** Schematic of DAPT treatment of human ALI epithelial cultures.
- **d.** Schematic of confocal image processing for DAPT-treated ALI cultures.
- **e.** *Left,* Box plots summarizing expression of genes uniquely upregulated in mature ciliated cells with smoking across ciliated cell subgroups and non-ciliated cells. *Right,* Box plots summarizing expression of genes uniquely downregulated in hybrid secretory/early ciliating cells across ciliated cell subgroups and non-ciliated cells. From left to right, the number of cells per box plot are 41, 62, 1,121, 679, 13,413, and 14,922. Box centers give the median, upper and lower box bounds correspond to first and third quartiles, and the upper/lower whiskers extend from the upper/lower bounds up to/down from the largest/smallest value, no further than 1.5 x IQR from the upper/lower bound (where IQR is the inter-quartile range). Data beyond the end of whiskers are plotted individually. Numbers above plot pairs are p-values for tests of mean difference based on two-sided Wilcox tests.

Supplementary Fig. 9

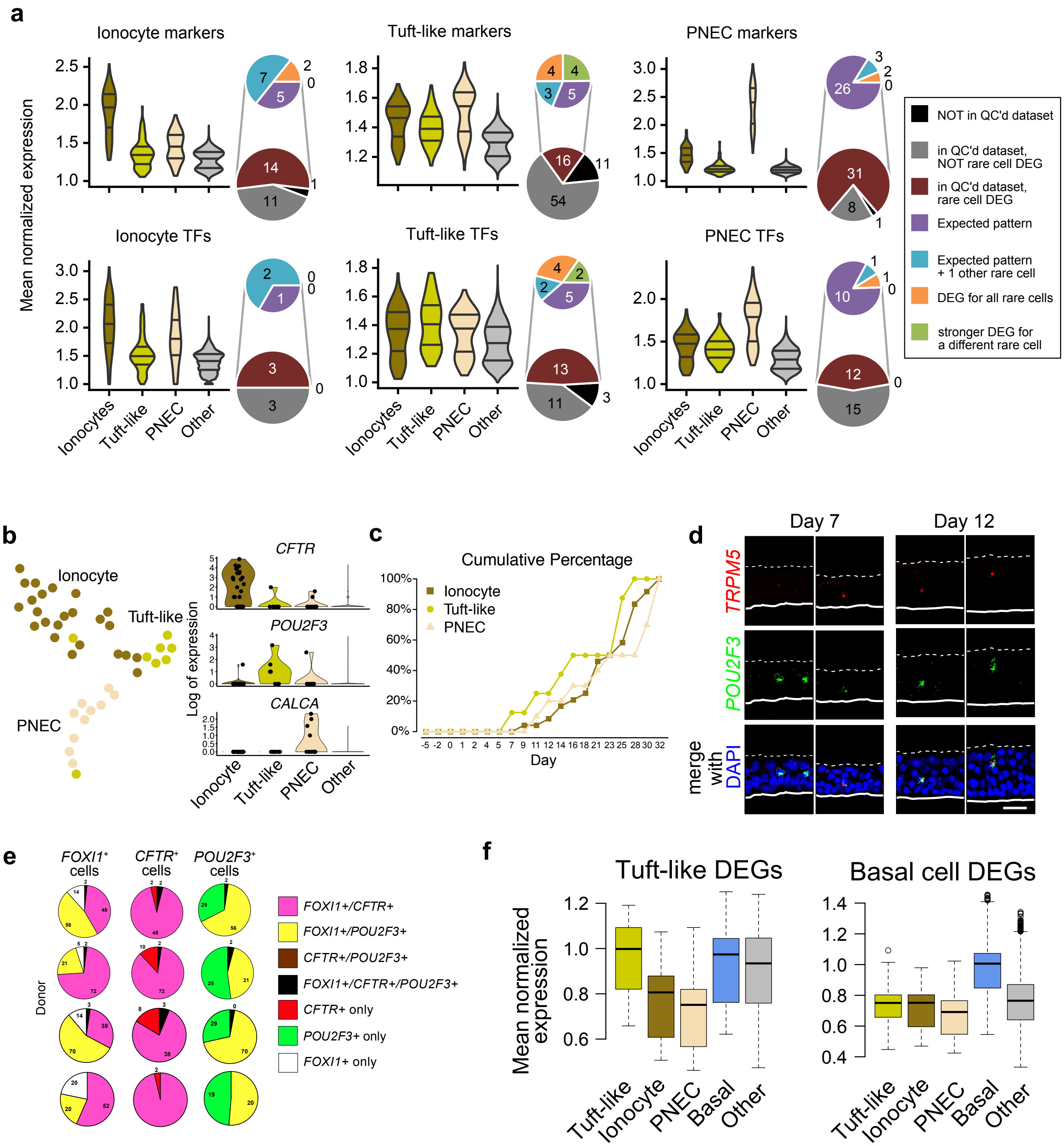

Supplementary Fig. 9 (with Fig. 7): *In vivo* human rare cell populations - comparisons with the literature and their characterization within *in vitro* cultures

a. Violin plots of average expression of recently published PNEC, ionocyte and tuft cell RNA markers and TFs[4] (n = 101, 92, 87, and 35,968 cells for ionocytes, tuft-like, PNEC, and other cells, respectively). From top to bottom, lines within violins give the third, second, and first quartiles. Pie charts to right of each violin plot specify proportions of genes that are present in our dataset, follow the specific expression pattern presented by Montoro et al. (2018) (i.e. only present in the rare cell substate indicated), or follow an alternative pattern as indicated. Also see Source Data for the genes that fall into these categories.

b. tSNE plot depicts SNN subclustering of rare cells found in ALI cultures of human tracheal epithelial cells across all 20 time points in this study. Violin plots of ionocyte, tuft, and PNEC markers identify the three substates.

c. Cumulative expression of ionocytes, PNECs and tuft-like cells captured by single cell sequencing at each time point in ALI culture.

d. FISH localizes tuft-like cells to early time points in *in vitro* culture through co-localization of *POU2F3* (green) and *TRPM5* (red) mRNAs. DAPI labeling of nuclei (blue). Dashed and solid lines represent the apical edge and basement membrane of the epithelium, respectively. Scale bar = 25 μm.

e. Donor variation of *CFTR*, *FOXI1*, and *POU2F3* FISH quantification. Number of cells is indicated in each pie.

f. *Left,* DEGs that distinguish Tuft-like cells from other rare cells have basal character compared to other rare cells and non-basal cells. The mean of all significant DEGs is plotted. *Right,* Tuft-like cells carry the most basal cell signature of all the rare cell types. The mean of the top 100 most upregulated genes in each of the differentiating and differentiating basal populations (compared to all other populations in the dataset) is shown. For box plots, n = 92, 101, 87, 15,371, and 20,597 for tuft-like, ionocyte, PNEC, basal, and other cells, respectively. Box centers give the median, upper and lower box bounds correspond to first and third quartiles, and the upper/lower whiskers extend from the upper/lower bounds up to/down from the largest/smallest value, no further than 1.5 x IQR from the upper/lower bound (where IQR is the inter-quartile range). Data beyond the end of whiskers are plotted individually.

Supplementary Fig. 10

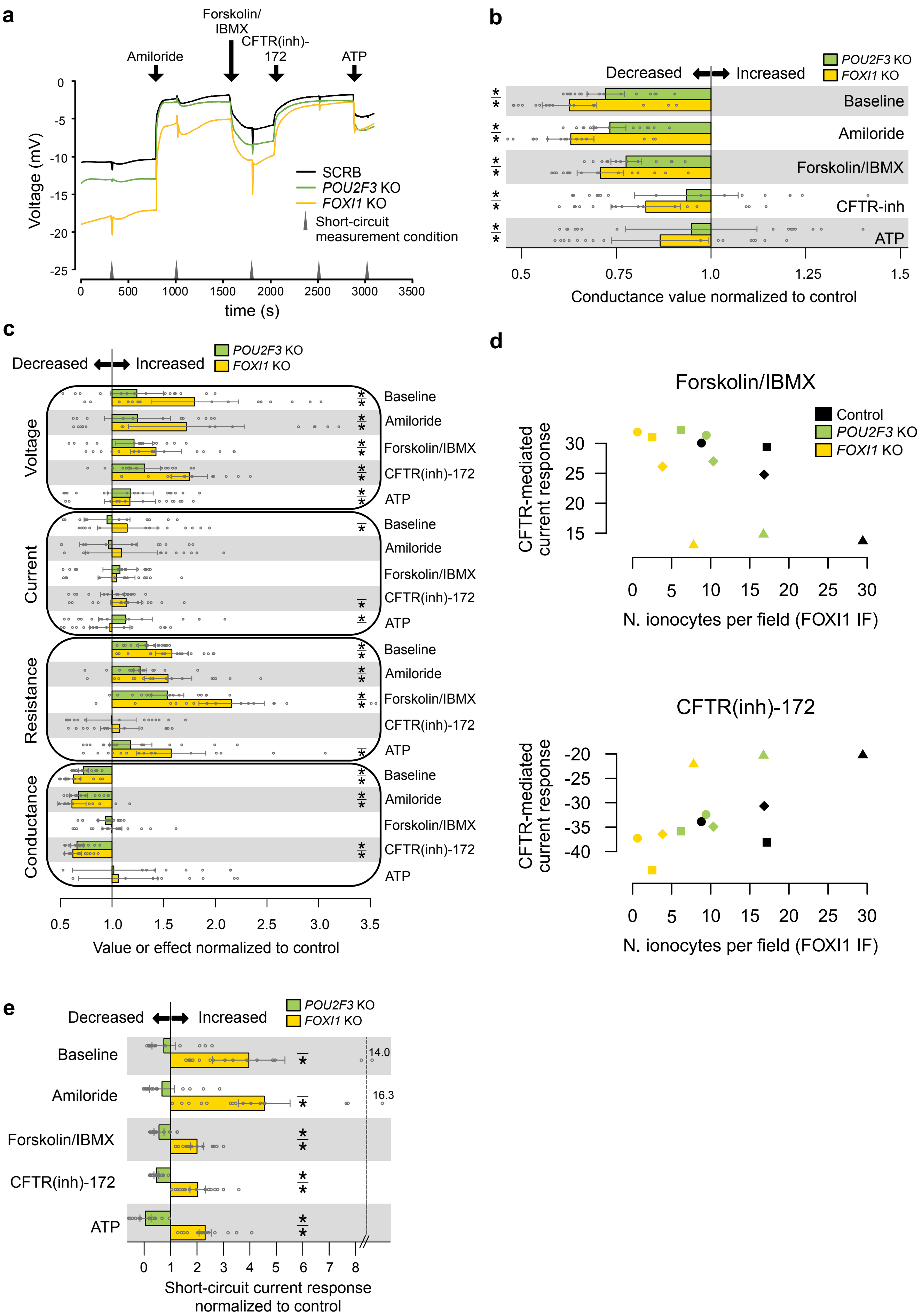

Supplementary Fig. 10 (with Fig. 8): Ussing chamber analysis of CRISPR-Cas9-edited human ALI cultures

a. Representative voltage traces for Ussing analysis. Stimulations are indicated, as well as intervals of short-circuit current measurement with pulsing to obtain resistance and conductance values (grey wedges). Voltage values of "0" obtained during short-circuit condition have been omitted. Stabilized values for each parameter (voltage, current, resistance and conductance) were collected at baseline and under each stimulation in four replicate inserts per KO for each of four donors at ALI Day 32.
b. Both KO cultures sustained the pattern of similar short-circuit current measurements with lower conductances relative to control cultures across a standard series of active transport manipulations, suggesting paracellular transport or "shunt" is affected by ionocyte-depletion. However, the difference in conductance between KOs and control cultures decreases as active transporters are inhibited toward the end of the series, indicating that active transport is also affected. Bars give estimated parameter values (normalized to control cultures) across four donors (four cultures each). Estimates correspond to the coefficient from a linear model, with donor treated as a random predictor variable. Points show individual measures, normalized to control estimates, and lines show standard error from the estimate. *, $p < 0.05$, when compared to control using an F-test with Satterthwaite approximation of degrees of freedom (exact p-values, top to bottom: 7.11e-9, 1.37e-11, 1.97e-9, 7.23e-13, 4.73e-9, 1.91e-11, 0.0228, 1.99e-8, 0.0132, 1.33e-7).
c. The effect of each stimulation on electrophysiological parameters was calculated as the difference between the stabilized value with stimulation and the stabilized value for the previous stimulation (or baseline for amiloride). These differences for each KO were then normalized to the average differences observed in control cultures. Bars give estimated parameter values (normalized to control cultures) across four donors (four cultures each). Estimates correspond to the coefficient from a linear model, with donor treated as a random predictor variable. Points show individual measures, normalized to control estimates, and lines show standard error from the estimate. *, $p < 0.05$, when compared to control using an F-test with Satterthwaite approximation of degrees of freedom (exact p-values, top to bottom, *voltage*: 0.0157, 1.72e-10, 0.0274, 4.39e-8, 4.98e-4, 2.50e-9, 3.85e-5, 8.47e-17, 0.00810, 0.0113; *current*: 0.289, 0.00224, 0.520, 0.101, 0.0605, 0.282, 0.893, 0.00281, 0.0208, 0.701; *resistance*: 9.63e-8, 5.38e-14, 0.00612, 8.73e-7, 1.22e-4, 1.58e-11, 0.913, 0.184, 0.117, 7.31e-6; *conductance*:

1.66e-10, 2.91e-14, 1.38e-5, 6.11e-7, 0.252, 0.987, 9.77e-10, 5.35e-11, 0.734, 0.239).

d. FOXI1⁺ nuclei counts vs CFTR-mediated current response in ALI Day 32 control, *POU2F3* KO, and *FOXI1* KO cultures. *Top*, effect of Forskolin/IBMX treatment on short-circuit current vs. $FOXI1^+$ nuclei by IF (see Figure 7h). *Bottom*, effect of CFTR(inh)-172 treatment on short-circuit current vs. $FOXI1^+$ nuclei by IF. For variance around each value, see Supplementary Figure 11b (CFTR-mediated current response) or Figure 7h ($FOXI1^+$ nuclei). Shape specifies cultures from the same donor.

e. The short-circuit current response during voltage clamping was calculated as the difference between the current value 2-4 seconds after voltage clamping and the current value immediately before clamping. Differences for each KO insert were normalized to the control insert run in the same batch on the same Ussing apparatus, such that the interval post-clamp was identical for each KO/control pair. Bars give estimated parameter values (normalized to control cultures) across four donors (four cultures each). Estimates correspond to the coefficient from a linear model, with donor treated as a random predictor variable. Points show individual measures, normalized to control estimates, and lines show standard error from the estimate. *, $p < 0.05$, when compared to control using an F-test with Satterthwaite approximation of degrees of freedom (exact p-values, top to bottom: 0.0550, 2.09e-4, 0.410, 5.36e-4, 8.79e-4, 1.56e-6, 5.69e-5, 3.92e-6, 2.08e-6, 8.44e-7).

Supplementary Fig. 11

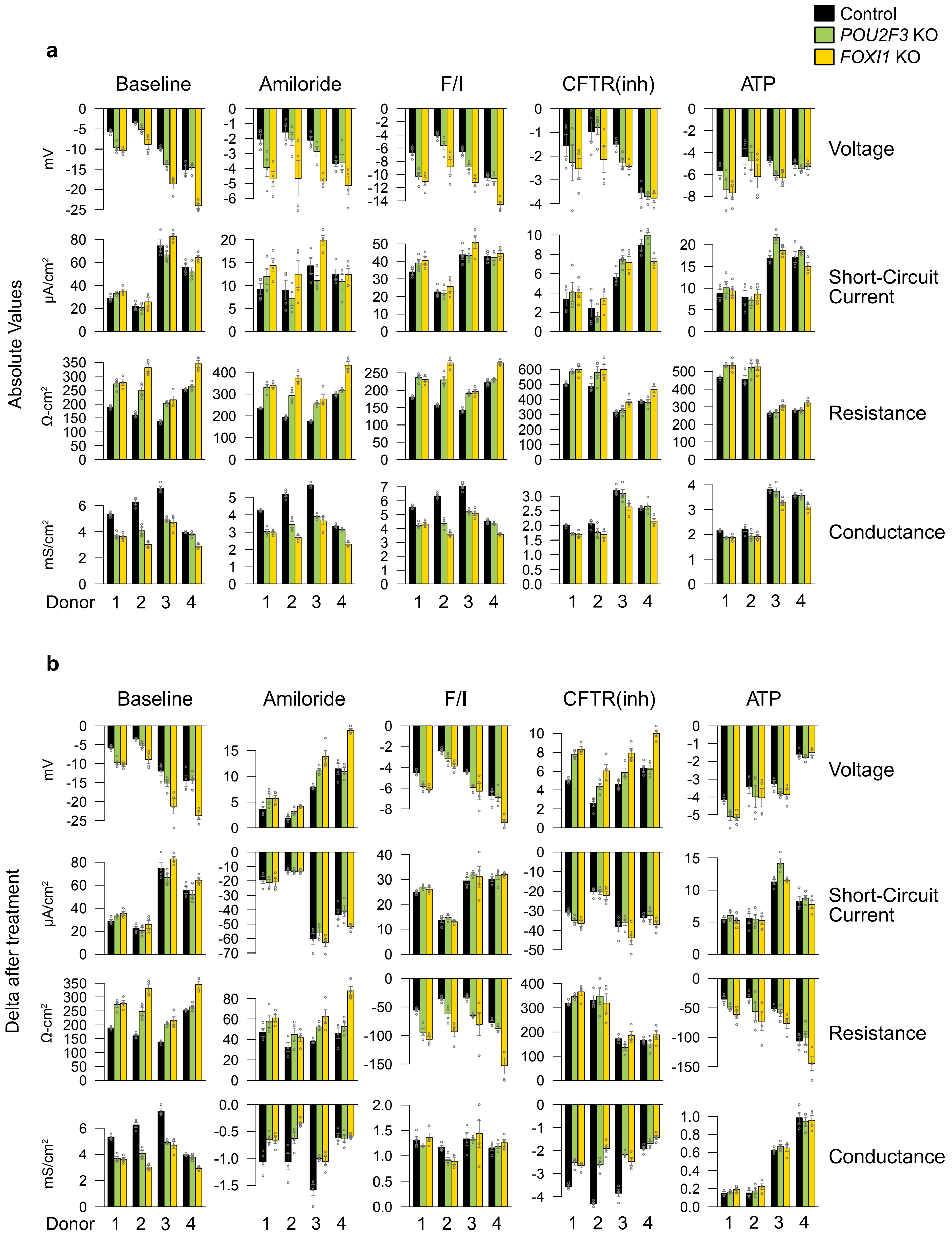

Supplementary Fig. 11 (with Fig. 8): Non-normalized Ussing chamber data of CRISPR-Cas9-edited human ALI cultures

- **a.** Stabilized raw data (absolute values) for voltage, short-circuit current, resistance and conductance (plot rows) for each step along the stimulation series demonstrated in Supplementary Fig. 10a (plot columns). Each set of bars shows the average parameter value across four replicate inserts for each trio of treatments (control, *POU2F3* KO, and *FOXI1* KO) and for each of four donors. Points show raw values and error bars give the standard error across the four replicates.
- **b.** Stabilized delta data for voltage, short-circuit current, resistance, and conductance (plot rows) for each step along the stimulation series demonstrated in Supplementary Fig. 10a (plot columns). The effect of each stimulation on electrophysiological parameters was calculated as the difference between the stabilized value with stimulation and the stabilized value for the previous stimulation (or baseline for amiloride). Baseline raw data are provided for context in the first column. Each set of bars shows the average delta value across four replicate inserts for each trio of treatments (control, *POU2F3* KO, and *FOXI1* KO) and for each of four donors. Points show raw delta values and error bars give the standard error across the four replicates.

Supplementary Table 1: Demographic information for human tracheal tissue samples

Donor	Age	Sex	Respiratory diseases?	Smoker Status	Total tobacco use (pack/day)	Years smoked	Years quit	Total pack years	CXR	PaO2	FiO2	other	Which study *
T89	10	F	N	Never									sc, TC, KO1
T137	27	M	?	Never					clear	330	100%		sc
T126	35	F	?	Never					clear except basilar atelectasis	111	30%		sc
T153	38	M	N	Never					clear	622	100%		sc
T165	64	M	N	Never					right lung clear				sc, KO2
T164	66	M	N	Never					basilar atelectasis	278	100%	COD stroke	sc
T166	68	F	N	Never					clear	370	100%		sc
T84	22	M	childhood Asthma	Light	1.5	2	2						sc, TC
T121	23	F	?	Light	0.5	unknown			mild pulmonary edema	100	60%		sc
T90	44	M	N	Heavy	1	30		30					sc
T91	54	M	N	Heavy	0.5-1	30		22.5					sc
T101	55	M	N	Heavy	1	25		25					sc
T120	57	F	N	Heavy	3	>30		90				skin cancer	sc
T85	59	M	N	Heavy	0.5	30		15					sc, TC
T154	61	M	?	Heavy	1.5	40		60	clear	408	100%		sc
T160	61	F	N	Heavy	0.5-1	30		22.5	L basilar atelectasis vs consolidation, R clear	206	100%		sc
T167	66	F	?	Heavy	1	40		40	perihilar congestion	313	100%	Bronch negative for infection	sc
T133	17	F	N	Unknown					tiny perihilar infiltrate vs atelectasis	427	100%		KO2, DAPT
T138	36	F	?	Never					clear	442	100%		KO2
T139	76	F	?	Never					possible apical scarring. Otherwise clear.	214	40%		KO2, DAPT
T147	69	M	?	Never					clear	127	30%		KO2

*sc = single cel RNA-seq, TC = time-course study, KO1 = *FOXN4* knock-out study, KO2 = *FOXI1/POU2F3* KO study, DAPT = hybrid cell validation study

Supplementary Table 2: Overlapping DEGs among *in vivo* rare cells

Average expression<br>Ionocyte Tuft-like PNEC other	Number of significant genes	Key genes & pathways	Potential Regulators
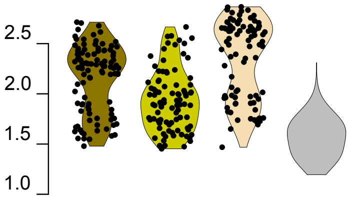	133	*ATP6V1A ATP6V0B ATP2A3 ATPB6V0E2 BIK CHD6 CHD7 CORO7 ENC1 FBXO32 H2AFY HACD3 ISG20 MSI2 QSOX1 REPIN1 RBP1 SORL1 SRGAP1*<br>proton-transporting ATPase activity<br>cell adhesion molecules<br>phagosome<br>synaptic vesicle cycling<br>collecting duct acid secretion<br>iron uptake and transport	*KIDINS200 ZNF254 PBXIP1 ZKSCAN1 PATZ1 ARID1B SCAND1 TULP4 ZFHX3 SMARCB1*
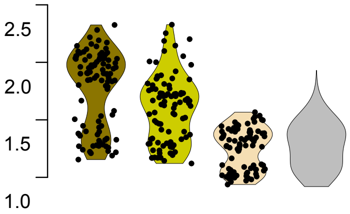	107	*CASC15 CDK14 CXXC5 DOCK4 FARP1 KLHL24 LDHB MACO1 MAP4K4 NDUFA9 NDUFS5 PTTG1IP SRM TBXAS1 TSPAN12 ULK1 WNK2*<br>ion transmembrane transporter activity	*LRCH4 SNF326 ARID2 REST FOXI1 ASCL3 FOXP1 DMRT2 TLE1 MAF SCAI SOX4 SOX9 PBX1 MXD1 TEAD2*
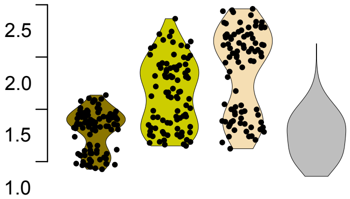	107	*ADA ADIRF ALAS1 ARVCF AZGP1 CCDC112 CRYM DUSP8 HYAL2 INPP5B NKL SLC39A6 SORT1 TBC1D16 TMEM108 TTC3 USP22*<br>unfolded protein response<br>protein processing in the ER<br>Wnt signaling	*ZNF195 ZNF3 CBX4 RSF1 JARID2 MLLT6 ST18 BTG1 ANKRD22 CBFA2T2*
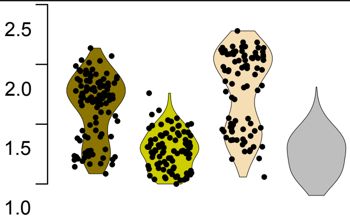	647	*ANK2 B2M CALM1 CAPN2 CD63 CISD1 HLA-A HLA-E KCNMA1 NEURL1 RFK SKAP1*<br>cholesterol biosynthesis<br>protein processing in ER<br>lysosome<br>TCA and electron transport chain<br>interferon signaling	*USF2 THAP12 ZMYND11 TCF4 NEO1 CRTC1 PURA GTF3A ARNT2 SREBF2 ZMIZ2*

Violin plots of shared gene signature expression levels, genes, and potential regulators across rare cell types implicate their relatedness.

References

1 Fischer, A. J. *et al.* Differential gene expression in human conducting airway surface epithelia and submucosal glands. *American Journal of Respiratory Cell Molecular Biology* **40**, 189-199 (2009).

2 Beane, J. *et al.* Reversible and permanent effects of tobacco smoke exposure on airway epithelial gene expression. *Genome Biol* **8** (2007).

3 Trapnell, C. *et al.* The dynamics and regulators of cell fate decisions are revealed by pseudotemporal ordering of single cells. *Nature Biotechnology* **32**, 381-U251, doi:10.1038/nbt.2859 (2014).

4 Montoro, D. T. *et al.* A revised airway epithelial hierarchy includes CFTR-expressing ionocytes. *Nature* **560**, 319-324 (2018).

5 Street, K. *et al.* Slingshot: cell lineage and pseudotime inference for single-cell transcriptomics. *BMC Genomics* **19**, 477-493 (2018).
