# Supplementary material for: Dissecting the cellular specificity of smoking effects and reconstructing lineages in the human airway epithelium
Source: Nat Commun. 2020 May 19;11:2485. doi: 10.1038/s41467-020-16239-z (PMC7237663; doi:10.1038/s41467-020-16239-z)
Supplement: Supplementary file 3 — Reporting Summary [file 41467_2020_16239_MOESM3_ESM.pdf]

nature research

Corresponding author(s): Max A. Seibold

Last updated by author(s): Mar 16, 2020

Reporting Summary

Nature Research wishes to improve the reproducibility of the work that we publish. This form provides structure for consistency and transparency in reporting. For further information on Nature Research policies, see Authors & Referees and the Editorial Policy Checklist.

Statistics

For all statistical analyses, confirm that the following items are present in the figure legend, table legend, main text, or Methods section.

n/a	Confirmed	
☐	☒	The exact sample size (*n*) for each experimental group/condition, given as a discrete number and unit of measurement
☐	☒	A statement on whether measurements were taken from distinct samples or whether the same sample was measured repeatedly
☐	☒	The statistical test(s) used AND whether they are one- or two-sided<br>*Only common tests should be described solely by name; describe more complex techniques in the Methods section.*
☐	☒	A description of all covariates tested
☐	☒	A description of any assumptions or corrections, such as tests of normality and adjustment for multiple comparisons
☐	☒	A full description of the statistical parameters including central tendency (e.g. means) or other basic estimates (e.g. regression coefficient) AND variation (e.g. standard deviation) or associated estimates of uncertainty (e.g. confidence intervals)
☐	☒	For null hypothesis testing, the test statistic (e.g. *F*, *t*, *r*) with confidence intervals, effect sizes, degrees of freedom and *P* value noted<br>*Give P values as exact values whenever suitable.*
☒	☐	For Bayesian analysis, information on the choice of priors and Markov chain Monte Carlo settings
☒	☐	For hierarchical and complex designs, identification of the appropriate level for tests and full reporting of outcomes
☐	☒	Estimates of effect sizes (e.g. Cohen's *d*, Pearson's *r*), indicating how they were calculated

Our web collection on statistics for biologists contains articles on many of the points above.

Software and code

Policy information about availability of computer code

Data collection	None
Data analysis	ImageJ version 1.51 was used for basolateral membrane quantification; Cell Ranger version 3.0 was used for in vivo single cell pre-processing; Cutadapt version 2.4 was used for trimming in vitro single cell reads; GSNAP version 2016_05_01 was used for mapping in vitro single cell reads; HTSeq-count version 0.8.0 was used for in vitro gene count quantification; DESeq2 version 1.16.1 was used to normalize AmpliSeq expression data; Seurat (R package) version 3.0.3.9015 was used for all single cell dataset integration, clustering, visualization, and differential expression analysis; heatmap3 (R package) version 1.1.1 used used for producing heat maps; Enrichr version 2017 (https://amp.pharm.mssm.edu/Enrichr/) was used for functional enrichment analysis; Monocle (R package) version 2.8 and Slingshot (R package) version 1.0 were used to construct single cell trajectories; Velocyto.R (R package) version 0.6 was used for estimating spliced/unsliced mRNA ratios; FGNet (R package) version 3.16.0 was used to construct functional gene network plots; custom code used has been deposited at github.com/seiboldlab/SingleCell_smoking.

For manuscripts utilizing custom algorithms or software that are central to the research but not yet described in published literature, software must be made available to editors/reviewers. We strongly encourage code deposition in a community repository (e.g. GitHub). See the Nature Research guidelines for submitting code & software for further information.

Data

Policy information about availability of data

All manuscripts must include a data availability statement. This statement should provide the following information, where applicable:

- Accession codes, unique identifiers, or web links for publicly available datasets
- A list of figures that have associated raw data
- A description of any restrictions on data availability

Gene lists and other source data associated with Figure and Supplementary Figure panels can be found in Supplementary Table 4. All raw and processed scRNA-seq

data used in this study have been deposited in the National Center for Biotechnology Information/Gene Expression Omnibus (GEO) with accession number GSE134174. It has been released as of Dec 11, 2019.

Field-specific reporting

Please select the one below that is the best fit for your research. If you are not sure, read the appropriate sections before making your selection.

☒ Life sciences ☐ Behavioural & social sciences ☐ Ecological, evolutionary & environmental sciences

For a reference copy of the document with all sections, see nature.com/documents/nr-reporting-summary-flat.pdf

Life sciences study design

All studies must disclose on these points even when the disclosure is negative.

Sample size	For single cell RNA sequencing, no formal sample size calculation was done. 15 tracheal donors were chosen to represent donor variability and this sample size represents one of the largest airway single cell data sets available to date. Confirming this, 15 tracheal donors were sufficient to define broad cell populations representative of the human trachea. The majority of our analyses was among the hundreds to thousands of cells for each cell type or state, which was plenty to detect hundreds of statistically significant differentially expressed genes. For the smoking analyses, we addressed donor variation by pooling cells for each cell type from 6 never smokers vs 6 heavy smokers, resulting in between 260 - 5473 cells for each smoke status for statistically significant differences. For the rare cell single cell analyses, 87 PNECs, 92 tuft-like, and 101 ionocytes were analyzed as this was the limited population in our dataset. Rare cell smoking trends were among never smoker:heavy smoker cell numbers of 29:46 (PNECs), 45:35 (tuft-like) and 40:56 (ionocytes).<br><br>For IF/ISH imaging (Figures 1defgh, 3d, 4g, 6adg, 7d, 8d, Supplementary Figures 1e, 2, 4, 6e, 9d), no sample size calculation was performed. Representative images are shown from at least 4 fields as specified in the Methods section, as is in concert with the standard in the field (e.g. Stubbs et al. 2012 Nat Cell Biol)<br><br>For the dense ALI time course (Figure 5, Supplementary Figure 7), no sample size calculation was performed. Basal cells from 3 donors were cultured and 2-4 inserts were harvested at 20 timepoints which accounted for donor and technical variation. The Ampliseq dataset included pooled inserts from a single donor of the three cultured. Results were consistent across donors and this sample size generated results in harmony with published epithelial biology.<br><br>For image quantitation, no sample size calculation was performed. Samples sizes were chosen in concert with standards in the field (e.g. Stubbs et al. 2012 Nat Cell Biol): (Figure 6bc) 584 control and 854 FOXN4 KO cells were analyzed from 5 fields across independent duplicate cultures of a single donor, (Figure 7d) epithelia from 8.4 cm of basolateral membrane at least 2 cm from each of 4 donors, (Figure 7h) at least 8 fields in 5 donors for each KO treatment, (Figure 7i) 8 fields in 3 donors for each KO treatment.<br><br>For the knock-out studies, no sample size calculation was performed. FOXN4 KO was performed two times with basal cells from a single donor. POU2F3 and FOXI1 KOs were performed in 4 (electrophysiology) or 5 donors (qPCR, imaging quantification). This sample size was sufficient to give consistent results.
Data exclusions	As stated in the manuscript clustering to determine cell types and marker genes included all 15 donors. Our single cell smoking analysis compared current, heavy smokers (n=6) to non-smokers (n=6), therefore we excluded a light smoker (<10 pack years smoking history), a former smoker, and a pediatric donor from this analysis. We also excluded non-epithelial cells, since epithelial cells were the focus of this manuscript (Supplementary Figure S1a).<br>For IF/ISH imaging, in-tact non-hyperplastic tissue was considered.<br>For Fig 7i, only the 3 donors with sufficient quality imaging were quantified.
Replication	The single cell RNAseq analysis was originally performed in 7 donors, and upon expanding our samples size to 15 donors, we largely replicated our findings. All attempts at replication of the experiments generated consistent results, with the exception of the POU2F3 KO in 1 of 5 donors. While this donor (T133) performed the same as the other 4 donors for the FOXI1 KO, and the POU2F3 KO had comparably reduced POU2F3 mRNA to the remaining donors, this KO performed like the wildtype in this donor for FOXI1, ASCL1, GRP mRNA, FOXI1+ nuclei, GRP+ cells and electrophysiology. This could be due to induction of an alternative pathway for ionocyte and PNEC production in this set of cultures, and/or age of the donor (the T133 donor was 17, compared to 36, 64, 69, 76). The wildtype-like response of this donor/KO was incorporated into the figures and statistics reported.
Randomization	Randomization was not relevant to our single cell RNAseq study, as we were not testing a treatment effect. Replicate ALI cultures were chosen at random for timepoint/endpoint harvests, and replicate aliquots of pooled single cell suspensions were randomly assigned to KO treatments.
Blinding	Our study results did not involve the combination of subjective decisions or a priori hypotheses, where classical blinding might be needed. Rather we conducted computational analyses and genome-wide statistical tests, then reported the results of these agnostic analyses. Therefore blinding was unnecessary. The electrophysiology was performed completely blinded to the donor/KO status.

Reporting for specific materials, systems and methods

We require information from authors about some types of materials, experimental systems and methods used in many studies. Here, indicate whether each material, system or method listed is relevant to your study. If you are not sure if a list item applies to your research, read the appropriate section before selecting a response.

Materials & experimental systems

n/a	Involved in the study
☐	☒ Antibodies
☐	☒ Eukaryotic cell lines
☒	☐ Palaeontology
☒	☐ Animals and other organisms
☐	☒ Human research participants
☒	☐ Clinical data

Methods

n/a	Involved in the study
☒	☐ ChIP-seq
☒	☐ Flow cytometry
☒	☐ MRI-based neuroimaging

Antibodies

Antibodies used

PRIMARY ANTIBODIES
chicken anti-KRT5 (BioLegend, cat# 905901)
mouse anti-TP63 (Santa Cruz, cat# sc8431)
rabbit anti-MKI67 (Abcam, cat# ab16667)
rabbit anti-KRT8 (Abcam, cat# ab81289)
mouse anti-MUC5AC (Invitrogen, cat# MA-38223)
rabbit anti-MUC5B (Santa Cruz, cat# sc-20119, clone H-300)
mouse anti-KRT14 (NeoMarkers, cat# MS-115-P, clone LL002)
rabbit anti-DMBT1 (Atlas Antibodies, cat# HPA040778)
mouse anti-SCGB3A1 (Thermo, cat# MA5-24147)
mouse anti-ACTA2 (Sigma, cat# A2547, clone 1A4)
rabbit anti-FOXN4 (Thermo, cat# PA5-62122)
mouse IgG2b anti-ac. alpha Tubulin (Sigma, cat# T6793)
rabbit anti-DEUP1 (CCDC67) (Sigma, cat# HPA010986)
mouse IgG1 anti-gamma Tubulin (TUBG1) (Sigma, cat# GTU88)
rabbit anti-SCGB1A1 (BioVendor, cat# RD1801022220-01, clone 410219)
mouse IgG1 anti-FOXJ1 (Thermo, cat# 14-9965-82, clone 2A5)
rabbit anti-FOXI1 (Atlas Antibodies, cat# HPA071469)
rat anti-ECAD (Thermo, cat# 13-1900)
mouse IgG1 anti-CFTR (Millipore, cat# 05-583)

SECONDARY ANTIBODIES
goat anti-rabbit IgG (H+L) (Invitrogen, cat# A11008)
goat anti-mouse IgG2b (Invitrogen, cat# A21242)
goat anti-mouse IgG1 (Invitrogen, cat# A21125)
donkey anti-chicken IgY Jackson (ImmunoResearch, cat# 703-545-155)
donkey anti-rabbit IgG (H+L) (Invitrogen, cat# A21207, A31573)
donkey anti-goat IgG (H+L) (Invitrogen, cat# A11058, A21447)
donkey anti-mouse IgG (H+L) (Invitrogen, cat# A21203, A31571)
donkey anti-rat IgG (H+L) (Jackson ImmunoResearch, cat# 712-585-153)

Validation

All primary antibodies were commercially available and validated for use in human samples as delineated below:

chicken anti-KRT5 is validated for IHC of human samples on the manufacturer's website, and clearly delineates airway basal cells by IF microscopy in this manuscript (e.g. Figure 1df, Figure 4g, Supp Figure 2, Supp Figure 4).

mouse anti-TP63 is validated for IF of human samples/cells via the manufacturer's datasheet, and displays nuclear signal in a subset of airway basal cells by IF microscopy in this manuscript (e.g. Figure 1d).

rabbit anti-MKI67 is validated for IHC and IF of human samples/cells on the manufacturer's website and displays nuclear signal in a subset of airway basal cells by IF microscopy in this manuscript (e.g. Figure 1d).

rabbit anti-KRT8 is validated by the Human Protein Atlas for IHC and IF of human samples/cells, and localizes to most non-basal cells in the human airway epithelium demonstrated in this manuscript (e.g. Figure 1f).

mouse anti-MUC5AC is validated for IHC and IF of human samples/cells by the manufacturer's datasheet, and localizes to the secretory granules of many secretory cells in this manuscript (e.g. Figure 1f, Figure 3d).

rabbit anti-MUC5B is now discontinued but has been validated for IF in human cultures/samples in numerous publications including PMID#25287927, and localizes to the secretory granules of many secretory cells in this manuscript (e.g. Figure 3d).

mouse anti-KRT14 is validated for IHC, ICC, and IF in human samples/cells, and localizes primarily to the SMG basal cells and a subset of surface basal cells in this manuscript (e.g. Figure 1h, Supp Figure 2, Supp Figure 6).

rabbit anti-DMBT1 is validated by the Human Protein Atlas for IHC, ICC and IF in human samples/cells, and specifically localizes to the SMG secretory cells in this manuscript (e.g. Supp Figure 1e, Supp Figure 6e).

mouse anti-SCGB3A1 is validated for ICC and IF of human samples/cells on the manufacturer's website, and specifically localizes

to the SMG secretory cells in this manuscript (e.g. Supp Figure 1e).

mouse anti-ACTA2 is validated for IHC and IF of human samples/cells on the manufacturer's website, and specifically localizes to the myoepithelial cells at the edges of SMG in this manuscript (e.g. Figure 4g).

rabbit anti-FOXN4 is validated for ICC, IF and IHC of human samples/cells on the manufacturer's datasheet, and localizes to early ciliating cell nuclei by IF confocal microscopy in this manuscript (e.g. Figure 6a).

mouse IgG2b anti-ac. alpha Tubulin is validated for IF, ICC and IHC of human samples/cells on the manufacturer's website and localizes specifically to the cilia of mature mulitciliated cells in wildtype cultures this manuscript (e.g. Figure 6ab)

rabbit anti-DEUP1 (CCDC67) is validated for IHC and IF by the manufacturer's website, and localizes specifically to deuterosomes in multiciliated cells in this manuscript (e.g. Figure 6d).

mouse IgG1 anti-gamma Tubulin (TUBG1) is validated for IHC and ICC in human samples/cells on the manufacturer's website, and specifically localizes to basal bodies in ciliated cells in this manuscript (e.g. Figure 6d).

rabbit anti-SCGB1A1 is validated for IHC in human samples according to the manufacturer's website, and localizes to a subset of secretory cells in this manuscript (e.g. Supp Figure 4b).

mouse IgG1 anti-FOXJ1 is validated for ICC, IF and IHC in human samples/cells according to the manufacturer's website, and specifically localizes to the nuclei of ciliated cells in this manuscript (e.g. Figure 7h).

rabbit anti-FOXI1 is validated by Human Protein Atlas for IHC and ICC-IF in human cells/samples, and specifically localizes to the nuclei of ionocytes or ionocyte-destined cells in this manuscript (e.g. Figure 7h).

rat anti-ECAD is validated for IF, IHC and ICC in human samples/cells via extensive publication on the manufacturer's website, and localizes to cell boundries in this manuscript (e.g. Figure 6g, Figure 7h).

mouse IgG1 anti-CFTR is validated for IHC in human samples/cells according to the manufacturer's website, exhibits severe depletion in CF patient-derived ALIs with the F508del genotype (EKV unpublished results), and concentrates to apical hotspots in cells with FOXI1+ nuclei along with various additional puncta in this manuscript (e.g. Figure 8d, Supp Data 1).

Eukaryotic cell lines

Policy information about cell lines

Cell line source(s)	NIH 3T3 Fibroblast cells were purchased from ATCC for use as feeder co-culture for primary human epithelial cells.
Authentication	No authentication was performed.
Mycoplasma contamination	No mycoplasma testing was performed.
Commonly misidentified lines (See ICLAC register)	No commonly misidentified lines were used.

Human research participants

Policy information about studies involving human research participants

Population characteristics	Tracheal tissue was obtained from smokers and non-smokers, ages 10-68. Full demographic details can be found in Supplementary Table 1. Smokers with at least 15 pack years were classified as "heavy", while smokers with fewer than 5 pack years were classified as "light".
Recruitment	Tracheal tissue was isolated from de-identified, deceased donors whose lungs were not suitable for transplant. Lung specimens were obtained from the International Institute for the Advancement of Medicine (Edison, NJ) and the Donor Alliance of Colorado. Smoking status was based on family-reported history, thus we chose strong criteria (>15 pack years vs never) as our cut-off for smoking to ensure smoking status was not incorrectly reported.
Ethics oversight	The National Jewish Health Institutional Review Board (IRB) approved the research under IRB protocols HS-3209 and HS-2240.

Note that full information on the approval of the study protocol must also be provided in the manuscript.
