# Supplementary material for: Dissecting the cellular specificity of smoking effects and reconstructing lineages in the human airway epithelium
Source: Nat Commun. 2020 May 19;11:2485. doi: 10.1038/s41467-020-16239-z (PMC7237663; doi:10.1038/s41467-020-16239-z)
Supplement: Supplementary file 4 — Description of Additional Supplementary Files [file 41467_2020_16239_MOESM4_ESM.pdf]

Description of Additional Supplementary Files

Supplementary Data 1

Key resources for experimental methods. This file contains reagent names, vendors and identifiers for all the experimental resources referred to in the Methods.

Supplementary Movie 1

Full z-stack of control Day 32 human ALI cultures illustrates FOXI1 (red) and CFTR (green) co-localization to nuclei and apical surface, respectively, of the same cells. ECAD (white) delineates cell boundaries. 4 slices appear in Figure 8d.
