# Supplementary figures and images for: Dissecting the cellular specificity of smoking effects and reconstructing lineages in the human airway epithelium
Source: Nat Commun. 2020 May 19;11:2485. doi: 10.1038/s41467-020-16239-z (PMC7237663; doi:10.1038/s41467-020-16239-z)

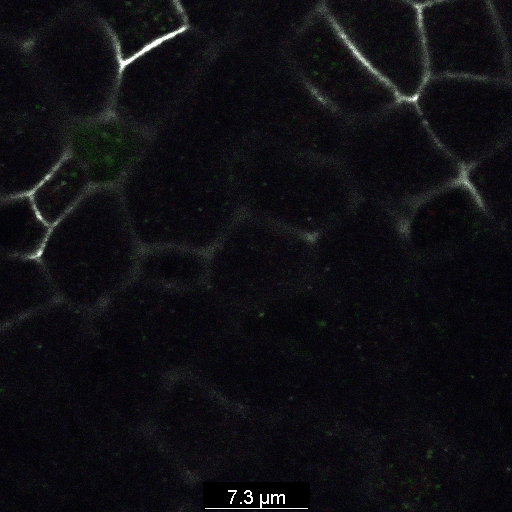

Supplement: Supplementary file 6 — Supplementary Movie 1 [file 41467_2020_16239_MOESM6_ESM.gif]
